# Fatty acid synthase expression and its association with clinico-histopathological features in triple-negative breast cancer

**DOI:** 10.18632/oncotarget.20152

**Published:** 2017-08-10

**Authors:** Ariadna Giró-Perafita, Ariadna Sarrats, Ferran Pérez-Bueno, Glòria Oliveras, Maria Buxó, Joan Brunet, Gemma Viñas, Teresa Puig Miquel

**Affiliations:** ^1^ New Terapeutics Targets Laboratory (TargetsLab), Department of Medical Sciences, University of Girona, Girona, Spain; ^2^ Pathology Department, Dr. Josep Trueta Hospital and Catalan Institute of Health (ICS), Girona, Spain; ^3^ Medical Oncology Department, Catalan Institute of Oncology (ICO), Girona, Spain; ^4^ Girona Biomedical Research Institute (IDIBGI), Girona, Spain

**Keywords:** EGFR, breast cancer molecular subtypes

## Abstract

Triple-Negative Breast Cancer (TNBC) has poor prognosis and no approved targeted therapy. We previously showed that the enzyme fatty acid synthase (FASN) was largely expressed in a small TNBC patients’ cohort and its inhibition synergized with cetuximab in TNBC preclinical mouse models. Here, we evaluated FASN and EGFR expression in a cohort of TNBC patients and we study their prognostic role and their association with clinico-histopathological features, intrinsic TNBC subtypes and survival.

FASN, EGFR, CK5/6 and vimentin expression were retrospective evaluated by Immunohistochemistry in 100 primary TNBC tumors. FASN expression was classified into high and low FASN groups. EGFR, CK5/6 and vimentin expression were used in TNBC intrinsic subtypes classification.

FASN was expressed in most of the TNBC patients but did not correlate with overall survival or disease-free survival in this cohort. High FASN group was significantly associated with positive node status. FASN expression was significantly higher in Basal-Like patients than in Mesenchymal-Like ones. EGFR expression was positive in 50% of the tumors, and those patients showed poorer DFS. Altogether, our findings provide a rationale for further investigation the prognostic role of FASN and EGFR expression in a larger cohort of TNBC patients.

## INTRODUCTION

Triple-negative Breast Cancer (TNBC) is a unique subset of breast cancer. Accounting for 15-20% of all breast carcinomas, it is characterized by the lack of the three most commonly targeted receptors in human breast cancer: the estrogen receptor, progesterone receptor, and human epidermal growth factor receptor 2 (HER2) [1, 2]. TNBC exhibits an aggressive clinical behavior and a high rate of local or distant relapses after treatment [2-4].

The introduction of DNA microarray technology defined initially four intrinsic subtypes of breast cancer, which were later extended to five by Perou *et al.*: Luminal A, Luminal B, HER2-enriched, Basal-like and Claudin-low or Mesenchymal-like [5-7]. In particular, the two major subtypes comprised in the TNBC are basal-like (BL) (∼50%) followed by the mesenchymal-like (ML) (∼30%) [8]. The stratification of TNBC patients may help in the election of appropriate treatments as each breast cancer subtype shows different incidence, survival and treatment response rates [8]. Currently, TNBC treatment relies solely on conventional chemotherapy. Research is focused into characterize TNBC with different molecular markers and find new therapy targets to improve TNBC patient’s outcomes [9].

Between 50-70% of TNBC express the epidermal growth factor receptor (EGFR) [7, 10], and its expression has been associated with poor prognosis [11]. Therefore, targeting EGFR initially seemed a feasible therapeutic strategy. Two completed trials investigated the addition of the monoclonal anti-EGFR antibody cetuximab to a platinum-crosslinking agent in metastatic TNBC [12]. In TBCRC001, the response rates of patients treated with cetuximab alone or in combination with carboplatin were relatively low at 6% and 17%, respectively [13]. The BALI-1 trial demonstrated that the addition of cetuximab to cisplatin increased overall response rate of TNBC patients from 10% to 20% [14]. Unfortunately, these combination treatments minimally increased progression-free survival and overall survival in TNBC suggesting that EGFR pathway may have alternate mechanisms of activation and prompted for the investigation of alternative therapeutic strategies for TNBC patients.

Lipogenic enzymes such as fatty acid synthase (FASN) are commonly overexpressed or show enhanced activity in neoplastic disease [15-17]. This enables long-chain fatty acids *de novo* synthesis essential for protein acylation, biological membrane synthesis, DNA synthesis and cell cycle progression of cancer cells [15, 16, 18]. We and others have reported that FASN inhibition (alone or in combination) induces apoptosis in several cancer cells and reduces the growth of human xenografts [19-24]. In this context, several reports highlight that FASN overexpression could be a putative biomarker and therapeutic target in several carcinomas, including breast cancers [23, 25-31].

We recently reported a specific expression of FASN in 29 core-biopsies from TNBC patients and we preclinical demonstrated that FASN inhibition could resensitize doxorubicin resistant cell lines. In addition, we showed strong synergism between FASN and EGFR inhibition in sensitive and doxorubicin resistant TNBC models, both *in vitro* and *in* animal TNBC models [19]. Here we evaluate FASN and EGFR expression in a cohort of TNBC patients and we study their prognostic role and their association with clinico-histopathological features, intrinsic TNBC subtypes and survival.

## RESULTS

### FASN expression and clinico-histopathological features of TNBC patients

**Figure 1:**
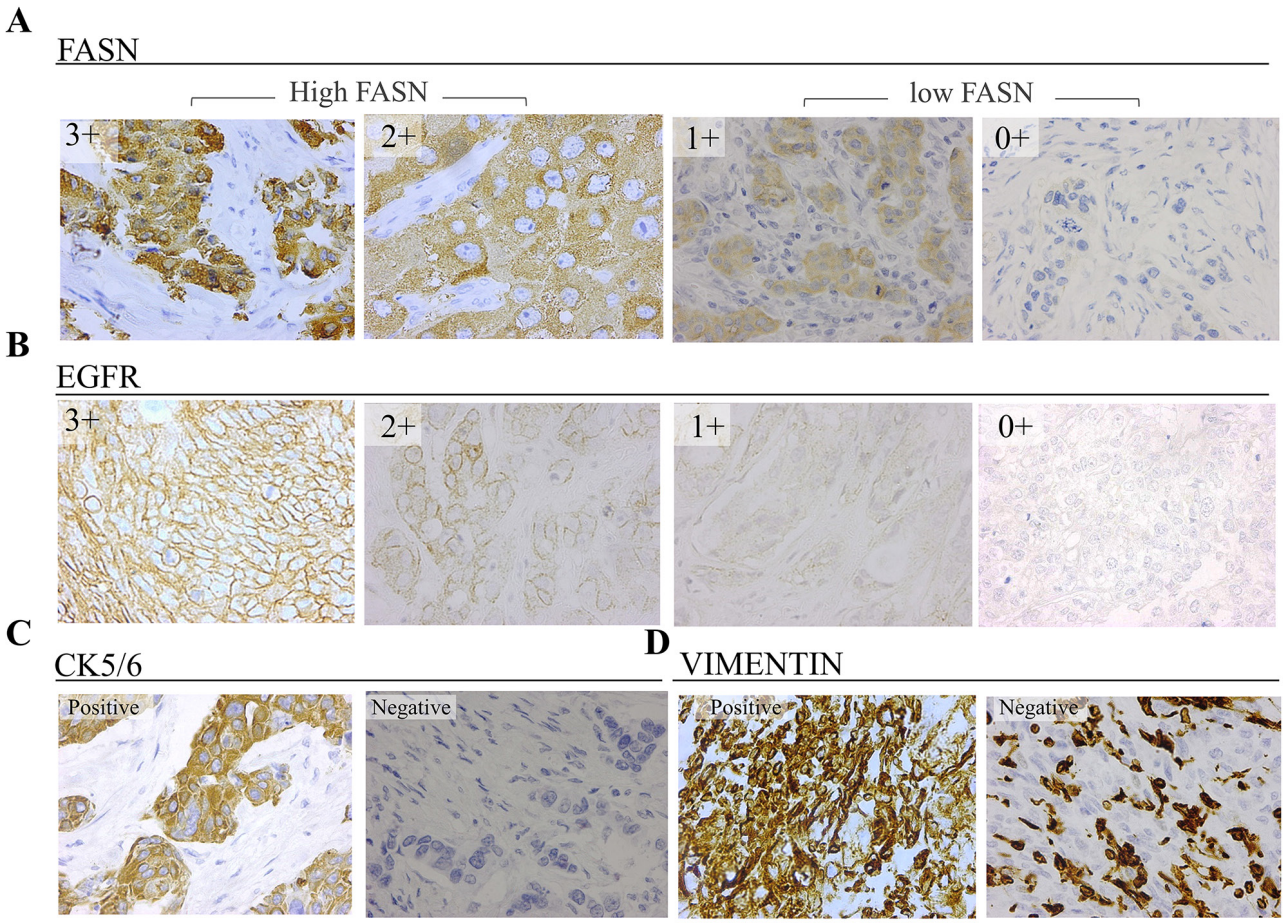
Representative immunostaining results of TNBC tissues (40X) for **(A)** FASN, with cytoplasmic localization, and **(B)** EGFR, with specific membrane staining **(C)** cytokeratin 5/6 with membrane and/or cytoplasmic localization **(D)** vimentin, with cytoplasmic staining.

A total of 100 women with primary Triple-Negative Breast Cancer (TNBC) diagnosed between 1990 and 2012 at Hospital Universitari Dr.Josep Trueta (Girona, Spain) were included in the study. Clinico-histopathological characteristics of the study group are shown in Table 1. FASN expression was determined by immunohistochemistry (IHC) in Tissue Microarray (TMA) of paraffin blocks of patients’ tumors sections. FASN expression was positive in almost all TNBC samples (92%). As described in methods section, patients were classified in low or high FASN expression according to staining intensity (Figure 1). High FASN expression was observed in 45% of TNBC samples. Interestingly, high FASN expression levels were significantly lower in non-tumoral tissues of the same patients being only detected in 22% of the patients (p<0.005).

**Table 1:** Clinico-histopathological characteristics according to FASN expression in TNBC

Characteristics	Total		Low FASN expression		High FASN expression		p-value*
	Value	%	Value	%	Value	%	
**Number of patients**	100		55	55.0%	45	45.0%	
**Mean age (yrs.)** ±SD	58.1	± 16.3	57.3	± 17.0	59.0	± 15.7	0.602*^1^
**Node status**							**0.038**
Negative	43	53.1%	28	63.6%	15	40.5%	
Positive	38	46.9%	16	36.4%	22	59.5%	
Unknown	19	-	11	-	8	-	
**Stage**							0.380
I	17	19.8%	11	23.4%	6	15.4%	
II	42	48.8%	24	51.1%	18	46.2%	
III	27	31.4%	12	25.5%	15	38.5%	
Unknown	14	-	8	-	6	-	
**Tumor grade**							0.050*^2^
I	0	0%	0	0.0%	0	0.0%	
II	11	17.5%	3	8.6%	8	28.6%	
III	52	82.5%	32	91.4%	20	71.4%	
Unknown	37	-	20	-	17	-	
**Ki-67**							1.000*^2^
≤ 20%	1	2.7%	1	5.0%	0	0.0%	
> 20%	36	97.3%	19	95.0%	17	100.0%	
Unknown	63	-	35	-	28	-	
Ki-67 (median; IQR)	(61.0	; 51.0)	(59.5	; 44.0)	(61.0	; 50.0)	0.460*^3^
**Surgery**							0.224
Lumpectomy	40	49.4%	19	43.2%	21	56.8%	
Mastectomy	41	50.6%	25	56.8%	16	43.2%	
Unknown	19	-	11	-	8	-	
**Adjuvant treatment**							0.168*^2^
Anthracyclines	6	11.1%	4	13.8%	2	8.0%	
Anthracy+Taxanes	43	79.6%	22	75.9%	21	84.0%	
Taxanes	3	5.6%	3	10.3%	0	0.0%	
Other	2	3.7%	0	0.0%	2	8.0%	
No treatment	12	-	5	-	7	-	
Unknown	34	-	21	-	13	-	

* Pearson’s Chi-Square Test.

*^1^ Unpaired t-Test

*^2^ Fisher’s Exact Test

*^3^ Mann-Whitney U-Test

There was no significant difference in patients’ mean age between low and high FASN expression groups, which was 57.3 and 59.0 years old respectively. There was a positive node involvement in 46.9% of the patients and it was significantly more frequent in the high expressing FASN group (59.5%) when compared to the Low FASN one (36.4%) (p=0.038). Approximately half of the patients (48.8%) were stage II, followed by stage III (31.4%) and stage I (19.8%). No association with FASN expression levels and stage was found. The most frequent tumor grade was III (82.5%) and a negative association between tumor grade and FASN levels was observed. Ki-67 was found to be > 20% in most of the patients (97%) therefore no association with high and low FASN expressing groups was observed.

### FASN expression and intrinsic subtypes of TNBC patients

We have recently reported that TNBC cellular models show different FASN expression levels [19]. In order to verify whether different levels of FASN were also observed in TNBC patients’ intrinsic subtypes, we stratified our study group in Basal-Like (BL), Mesenchymal- Like (ML) and Non-BL/Non-ML(NonBLML) as described in the Methods section according the expression of EGFR, cytokeratin 5/6 (CK5/6) and vimentin (Figure 1). The frequencies of each TNBC subtype in our cohort of TNBC were BL (56.1%), ML (31.7%) and NonBLML (12.2%) (Table 2).

**Table 2:** FASN expression in intrinsic subtypes of TNBC patients

	Total		Low FASN expression		High FASN expression		p-value
	Frequency	%^1^	Frequency	*%*^2^	Frequency	*%*^*2*^	
**Intrinsic subtypes**							<0.001*
Basal-Like	46	56.1%	22	*47.8%*	24	*52.2%*	] 0.020*^1^
Mesenchymal-like	26	31.7%	21	*80.8%*	5	*19.2%*	] <0.001*^1^
Non-BL/Non-ML	10	12.2%	1	*10.0%*	9	*90.0%*	
Unknown	18		11	-	7	-	
**Total**	**100**						

^1^ Percentage within the whole TNBC group

^2^ Percentage within each TNBC intrinsic subtype

* Fisher’s Exact Test

*^1^Adjusted p-values by Multiple comparisons Bonferroni method

Regarding FASN expression, BL patients showed similar percentages of low and high expression (47.8% vs 52.2%), while ML patients showed a marked predominant prevalence of low FASN expression (80.8%). In addition, most patients classified as neither Basal nor Mesenchymal demonstrate to express FASN at high levels (90.0%). The frequencies of low and high FASN populations were significantly different between BL and ML patients and also when comparing ML and NonBLML.

### EGFR, cytokeratin 5/6 and vimentin association with FASN expression in TNBC

EGFR, CK5/6 and vimentin are markers commonly expressed in TNBC which its expression has been associated with poor outcome [10, 32-35]. Here, we studied their individual association with FASN expression (Table 3).

**Table 3:** FASN association with other IHC markers in TNBC

	Total		Low FASN expression		High FASN expression		p-value*
	Frequency	%	Frequency	%	Frequency	%	
**EGFR**							0.095
Negative	44	**55.0**%	28	**62.2**%	16	**45.7**%	
Focal positive	2	**2.5**%	0	**0.0**%	2	**5.7**%	
1+	24	**30.0**%	14	**31.1**%	10	**28.6**%	
2+	3	**3.8**%	1	**2.2**%	2	**5.7**%	
3+	7	**8.8**%	2	**4.4**%	5	**14.3**%	
*(Total Positives)*	*(36)*	***(45%)***	*(17)*	***(37.8%)***	*19*	***(54.3%)***	
Unknown	20		10		10		
**Cytokeratin 5/6**							0.694
Negative	73	**73.0**%	41	**74.5**%	32	**71.1**%	
Focal positive	21	**21.0**%	11	**20.0**%	10	**22.2**%	
Positive	6	**6.0**%	3	**5.5**%	3	**6.7**%	
*(Total Positives)*	*(27)*	***(27.0%)***	*(14)*	***(25.5%)***	*(13)*	***(28.9%)***	
Unknown	0		0		0		
**Vimentin**							**<0.001**
Negative	23	**28.0**%	2	**4.5**%	21	**55.3**%	
Focal positive	23	**28.0**%	15	**34.1**%	8	**21.1**%	
Positive	36	**43.9**%	27	**61.4**%	9	**23.7**%	
*(Total Positives)*	*(59)*	***(72.0%)***	*(42)*	***(95.5%)***	*(17)*	***(44.7%)***	
Unknown	18		11		7		

* Mantel-Haenszel Test for linear Trend.

EGFR staining was positive in 45% of the samples, being 1+ the most abundant group of positivity (30%). Higher percentages and intensities of EGFR positive tissue samples were observed in the high FASN group compared to the Low one. However, a significant linear trend could not be determined. Only 27% of the patients of this study expressed CK5/6. This biomarker expression was not associated with FASN expression. Vimentin was found to be positive or focal positive in about 72% of the patients, and its expression was significantly inversely associated with the expression of FASN.

### Survival analysis

#### Survival analyses

**Figure 2:**
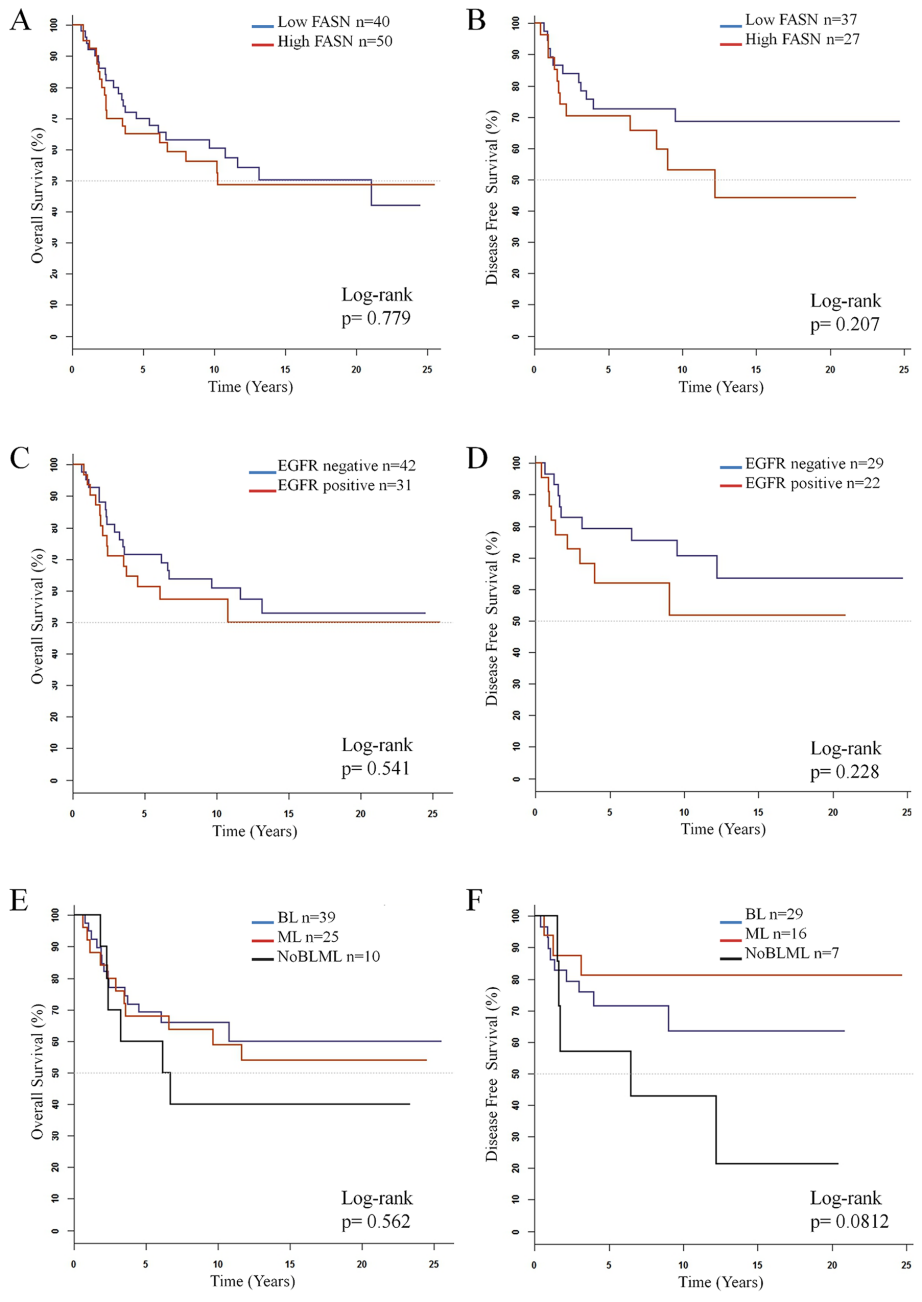
Kaplan-Meier estimate curves of overall survival (N=90) and disease-free survival (N=64) for **(A)** and **(B)** FASN, **(C)** and **(D)** EGFR and **(E)** and **(F)** molecular subtypes in TNBC patients.

The log-rank test showed no differences in Overall-Survival (OS) (N=90) or Disease-Free Survival (DFS) (N=64) between the Low and high FASN expressing groups (Figure 2A, 2B). Nevertheless, survival rates were usually lower in the high FASN group. For example, OS 3-year probability was 80% (95% CI: 69-91%) for patients with Low FASN levels and 70% (95% CI: 57-85%) for patients with high FASN. For DFS, 3-year probability was 83% (95% CI: 73-96%) for patients with Low FASN levels and 70% (95% CI: 55-90%) for patients with high FASN.

When considering EGFR expression, no differences in OS or DFS log-Rank test were observed between patients with positive expression and negative expression of this protein. However, survival rates were slightly higher for those patients with no expression of EGFR (Figure 2C, 2D).

Regarding intrinsic subtype classification, no differences were observed neither in OS outcome nor in DFS between subtypes (Figure 2E, 2F), although for this last parameter BL patients showed a 5-year probability of 71% (CI: 56-90%) while the probability for ML was 81% (CI: 64-100%). The NonBLML subtype showed the poorest OS and DSF, with values of 5-year probability of 60% (CI:53-89%) and 57% (CI:30-100%) respectively.

**Figure 3:**
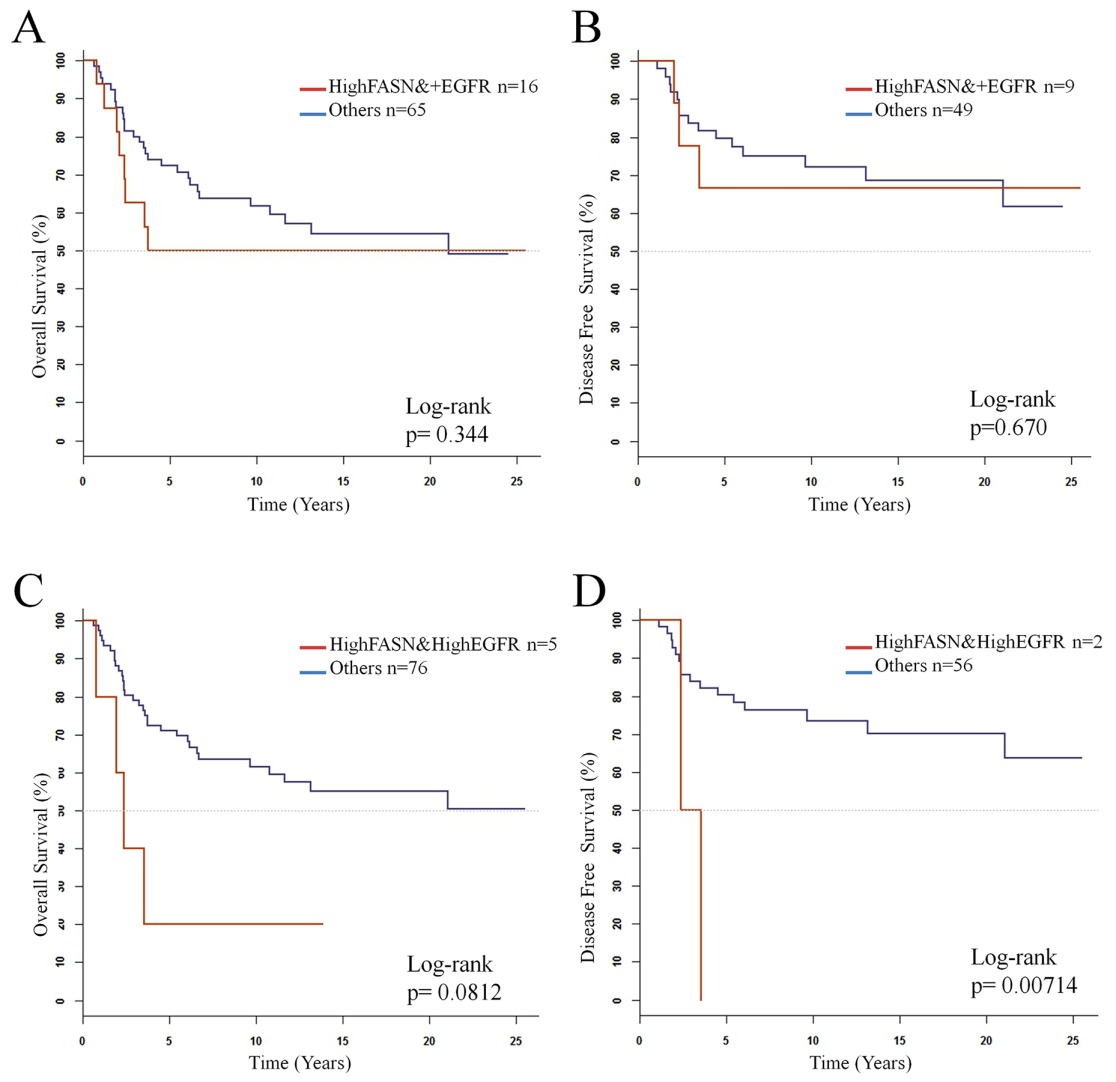
Kaplan-Meier estimate curves of overall survival (N=90) and disease-free survival (N=64) for **(A)** and **(B)** coexpression of highFASN and positive EGFR, **(C)** and **(D)** coexpression of high FASN and high EGFR.

While high FASN or positive EGFR expression alone did not show any significant association with survival, we decided to perform survival analysis of patients with combined high FASN expression and positive EGFR expression. As shown in Figure 3A, 3B, these patients show worse OS and DFS than the rest of the patients, however, this differences were still no significant. When focusing on the patients high co-expression of both FASN and EGFR (Figure 3C, 3D) both OS and DFS were significantly worse (p= 0.0812 and p= 0.00714, respectively). However, the conclusions driven from these analyses are hampered by the reduced samples sizes.

#### Cox analysis

In the Cox univariate regression analysis, age>49 years, advanced stage (III), and positive expression of CK5/6 were significantly associated with poor OS and DFS (Tables 4 and 5). Additionally, Positive node status and high EGFR expression (3+) were significantly associated with poor DFS. In contrast, FASN expression and Intrinsic TNBC Subtypes showed neither a significant association with OS nor with DFS. However, DFS HR was slightly higher for the high FASN expressing group (HR=1.69) when compared to low FASN one.

**Table 4:** Cox univariate analysis of overall survival

Overall Survival (N=90)				
Factor	N (n)^a^	HR^b^	(95% CI)^c^	p-value
**Fasn_2g**				
Low	50 (23)	1		
High	40 (19)	1.09	(0.59 -2.00)	0.780
**Stage**				
I	17 (3)	1		
II	38 (16)	2.55	(0.74 – 8.74)	0.138
III	25 (18)	8.75	(2.56 – 29.91)	**<0.001**
Unknown	10 (5)	3.30	(0.78 – 13.91)	0.104
**Age**				
≤ 49 years	29 (6)	1		
> 49 years	61(36)	3.73	(1.56 – 8.90)	**0.003**
**Intrinsic subtype**				
Basal-like	39 (14)	1		
Mesenchymal-like	25 (11)	1.09	(0.49 – 2.41)	0.829
Non-BL non-ML	10 (6)	1.65	(0.63 – 4.30)	0.305
Unknown	16 (11)	1.73	(0.78 – 3.82)	0.177
**Node status**				
Negative	41 (17)	1		
Positive	34 (18)	1.80	(0.92 – 3.51)	0.085
Unknown	15 (7)	1.25	(0.52 – 3.01)	0.624
**EGFR**				
Negative	42 (18)	1		
Focal positive	2 (1)	1.36	(0.18 – 10.23)	0.768
1+	21 (8)	0.98	(0.43 – 2.27)	0.967
2+	2 (1)	2.16	(0.28 – 16.42)	0.458
3+	6 (4)	2.56	(0.86 – 7.63)	0.092
Unknown	17 (10)	1.46	(0.67 – 3.17)	0.337
**Vimentin**				
Negative	22 (12)	1		
Focal positive	22 (7)	0.52	(0.21 – 1.33)	0.175
Positive	30 (12)	0.58	(0.26 – 1.30)	0.186
Unknown	16 (11)	1.04	(0.46 – 2.37)	0.928
**Cytokeratin 5/6**				
Negative	70 (35)	1		
Focal positive	15 (3)	0.36	(0.11 – 1.16)	0.088
Positive	5 (4)	3.52	(1.21 – 10.28)	**0.021**

^a^ N= number of patients at risk; n= number of events

^b^ Hazard ratio

^c^ 95% confidence interval

**Table 5:** Cox univariate analysis of disease free-survival

Disease free survival (N=64)				
Factor	N (n)^a^	HR^b^	(95% CI)^c^	p-value
**Fasn_2g**				
Low	37 (11)	1		
High	27 (12)	1.69	(0.74 – 3.83)	0.212
**Stage**				
I	17 (3)	1		
II	25 (7)	1.61	(0.42 – 6.22)	0.491
III	19 (13)	8.21	(2.28 – 29.54)	**0.001**
**Age**				
≤ 49 years	28 (8)	1		
> 49 years	36 (15)	1.63	(0.69 – 3.85)	0.266
**Intrinsic subtype**				
Basal-like	29 (9)	1		
Mesenchimal-like	16 (3)	0.48	(0.13 – 1.78)	0.273
Non-BL non-ML	7 (5)	2.27	(0.76 – 6.82)	0.143
Unknown	12 (6)	1.38	(0.49 – 3.90)	0.547
**Node status (N=61)**				
Negative	35 (10)	1		
Positive	26 (13)	2.59	(1.12 – 5.99)	**0.026**
**EGFR (N=63)**				
Negative	29 (9)	1		
Focal positive	1 (1)	3.01	(0.38 – 24.01)	0.280
1+	16 (4)	0.96	(0.29 – 3.13)	0.945
3+	4 (4)	11.05	(3.05 – 40.00)	**< 0.001**
Unknown	13 (5)	1.35	(0.45 – 4.04)	0.589
**Vimentin**				
Negative	12 (7)	1		
Focal positive	17 (4)	0.39	(0.11 – 1.34)	0.135
Positive	23 (6)	0.33	(0.11 – 1.00)	0.050
Unknown	12 (6)	0.68	(0.23 – 2.04)	0.493
**Cytokeratin 5/6**				
Negative	47 (18)	1		
Focal positive	13 (2)	0.41	(0.09 – 1.75)	0.227
Positive	4 (3)	4.54	(1.28 – 16.10)	**0.019**

^a^N= number of patients at risk. n= number of events

^b^Hazard ratio

^c^95% confidence interval

## DISCUSSION

Triple-Negative Breast Cancer treatment is mainly restricted to a combination of anthracyclines and taxanes [36]. Despite several efforts, any effective target therapy has been yet approved for TNBC treatment. Therefore, there is an urge to identify novel molecular targets for this breast cancer subtype.

TNBC patients tend to be younger and have larger and higher grade tumors with more lymph node involvement compared with other breast cancer subtypes [4, 37, 38]. Accordingly, our TNBC cohort has large tumors (48.8% stage II and 31.4% stage III) with high histologic grade (82,5% grade III), high Ki-67 proliferation index tumors (>20%; 97.3%) and high positive nodal involvement (46.9%).

Fatty acid synthase (FASN) has arisen as a potential target in different types of cancer and even currently a FASN inhibitor is being evaluated in a clinical trial [39]. In a recent study with a small cohort of TNBC patients, we have reported a positive FASN staining in all TNBC samples analyzed and 31% with high FASN positivity [19]. In the present study, we have extended these results using a large cohort of patients (n=100). FASN was positive in 92% of the tumor tissue samples and 45% of them showed high FASN levels.

When analyzing FASN association with clinical characteristics, we found that high FASN expression was associated with positive node involvement. The identification of tumor cells in lymph nodes had become a standard protocol in the clinic, as it has been proved to be one of the most powerful markers to predict patient outcome [40]. Several studies have found FASN as a poor prognosis marker in cancers such as in lung [41], ovarian [26], gastric [42] or in early breast carcinomas patients [25] among others. Furthermore, its role in drug resistance (and so relapse) has been identified in several pre-clinical studies [19, 43]. The long-rank test in our cohort of patients showed a markedly tendency (without being significant p=0.207) in poor DSF in patients with high FASN expression compare to the low FASN expressing group. The patient cohort size (OS n=90, DFS n=64), the fact that 90% of samples included in the study were positive for FASN, and that TNBC is itself an aggressive subtype of BC might be some of the limitation in the OS and DFS analysis in this study.

The molecular classification of breast cancer provided new insight in its biology. The two major subtypes identified within the Triple-negative breast cancers, basal-like (BL) and mesenchymal-like (ML) showed variations in growth rate, cellular composition and clinical outcomes compared to other molecular subtypes of breast cancer [6, 44]. Using IHC for EGFR, CK5/6 and vimentin we classified our cohort of patients in Basal-Like (56.1%), Mesenchymal-Like (31.7%) and Non-Basal/Non-Mesenchymal like (12.2%), obtaining ratios similar to the ones observed in other cohorts of patients [6, 8]. Interestingly, we found that FASN expression levels were significantly higher in BL patients than in ML, in agreement with our previous results *in vitro* [19]. BL breast cancer has been described to be a highly-proliferative subtype, contrary to the ML which show low expression of the proliferative and luminal gene cluster and is enriched with EMT markers [6, 8, 45]. Accordingly, FASN expression was negatively associated with tumor grade and vimentin expression, both characteristics described to be closely associated with ML tumors [6, 46-48]. Furthermore, the association between FASN and proliferation and its role in tumor growth suppression due to its inhibition has already been observed both *in vivo* and *in vitro* in several carcinomas [19, 20, 28]. Interestingly, poor DFS seem to correlate with FASN expressing levels when analyzing patients’ outcomes regarding its molecular classification (Table 2: high FASN % in ML=19,2%, BL= 52,2% and NonBL/nonML= 90%; p≤0.001).

We also analyzed the cytokeratins 5/6 and vimentin used to classify our patients in TNBC intrinsic subtypes. Cytokeratins 5/6 were positively immunostained in 27% of the patients and their expression was significantly associated with poor OS and DFS in concordance with previous studies [10, 33]. On the other hand, vimentin, a marker already described to be enriched in TNBC and associated with poor outcome [32, 35], was positive in 72% of the patients. Both ML and BL molecular subtypes are representative of poor outcomes subtypes [6].

EGFR expression is a poor prognosis marker frequently expressed in TNBC [34, 47]. In our patients set, EGFR staining was positive in 45% of the tumor samples. In agreement, other authors have shown that between 50-70% of TNBC patients are EGFR positive [7, 10]. DFS and OS for EGFR expression confirmed the correlation between EGFR positivity and poor survival observed in other studies [11, 48], although the differences were not significant in our study probably due to the limited number of patients. Interestingly, the univariate analysis showed that high EGFR expression (3+) was significantly associated with poor DFS. Interestingly, when considering the patients with high co-expression of both EGFR and FASN but also patients EGFR+ with high FASN levels significant differences in both OS and DFS could be observed. Although these survival analyses should be revaluated in a larger cohort of patients, they are bringing up the possible relevance of these to co-markers in TNBC patients. In fact, we have shown in an earlier preclinical study a strong synergism between FASN inhibitors and cetuximab at low concentrations in Basal-Like (BL) and Mesenchymal-Like (ML) cell lines and in TNBC orthoxenografts, without signs of toxicity [19]. EGFR inhibitors have been evaluated in combination with common chemotherapy, although most of the clinical trials have failed in order to improve significantly OS or DFS. The results obtained in this study may be, however, limited by the fact that the samples were retrospectively analyzed and also by the number of patients evaluated. Therefore, future prospective studies with a larger cohort of patients should be carried out.

In summary, we found FASN was expressed in most of the TNBC patients. Although FASN did not correlate with overall survival or disease-free survival in this cohort, high FASN expressing patients showed a marked tendency in lower OS or DFS rates in comparison with low FASN patients. FASN high expression was significantly associated with positive node status, one of the most powerful markers for predicting relapse. FASN expression was also significantly higher in Basal-Like patients than in Mesenchymal-Like ones. EGFR expression was positive in 45% of the tumors, and those patients showed poorer DFS. We have previously shown in a preclinical setting that the co-treatment of FASN and EGFR could be an effective therapeutic strategy for TNBC. Altogether, our findings provide a rationale for further investigation of the prognostic role and predictive biomarker of FASN and EGFR expression in TNBC.

## MATERIALS AND METHODS

### Patients’ selection, tissue samples and assessments

The study group consisted of 100 patients with primary Triple-Negative Breast Cancer (TNBC) diagnosed between 1990 and 2012 at Hospital Universitari Dr.Josep Trueta (Girona, Spain).

For each patient, clinical and histopathological feature were obtained from medical records: age, stage, surgery, chemotherapy, relapse, histological grade, lymph node involvement and Ki-67 grade. Stage was determined according TNM classification (7th edition of AJCC cancer staging manual [49]). Histological grade was defined using Bloom-Richarson grading system. FASN, cytokeratins 5/6, EGFR and vimentin expression were evaluated on tissue microarrays (TMA) containing tissue sections of patients’ primary tumor obtained by surgery. Analysis was carried out by two board-certified pathologists. The protocol was approved by the Institutional Review Board of Dr. Josep Trueta Hospital and an informed written consent was obtained from the patients included in the study.

### Construction of tissue microarrays (TMA)

From each patient’s tissue block, four tumoral cores and one non-tumoral peripherical core of 1mm were extracted and placed into an 8×5 recipient block. Each TMA contained 8 blocks, each one from a different patient. All samples were histologically reassessed by the pathologist to verify tumoral and non-tumoral spots before IMC analysis. All tumor spots included in the study contained more than 50% tumor cells.

### Immunohistochemistry on TMA

Immunohistochemical staining was performed on formalin-fixed, paraffin-embedded tissue TMA sections. Briefly, 3 μm-thick TMA tissue sections were placed onto adhesive slides and treated with the PT link (DAKO) solution. Immunohistochemical staining was performed using the following primary antibodies: anti-Fatty Acid Synthase polyclonal antibody (1:100, Enzo Life Sciences), anti-EGFR monoclonal antibody (1:100, clone D38B1, CellSignaling), anti-Cytokeratin 5/6 monoclonal antibody (1:50/100, Clone D5/16 B4, DAKO) anti Monoclonal Mouse Anti-vimentin (1:100/200, Clone Vim 3B4, DAKO). Sections were washed with PBS and sequentially incubated at room temperature for 45 minutes with antirabbit or antimouse IgG. Immunodetection was performed with the kit EnVision™ (DAKO, Glostrup, Denmark) using the AutostainerPlus Link (DAKO).

### Interpretation of immunohistochemical staining

The status of all immunohistochemical markers was determined by using light microscopy to assess the proportion and intensity of stained cells. FASN staining was considered positive when staining of >10% of the tumor cells, and the intensity was scored from 0 to 3: 0, no staining; 1, low staining; 2, moderate staining; and 3, high staining and was determined both in tumoral spots and non-tumoral, peripherical tissue. For analytical purposes patients with 0-1+ FASN staining were grouped as low-FASN expression and patients with 2+-3+ FASN staining were grouped as High-FASN expression. EGFR staining was evaluated as positive when staining of >1% of the cells (membrane stained or both membrane and cytoplasm) or negative (only cytoplasm stained) [35]. When positive, EGFR expression was scaled from 1+ to 3+. High EGFR expression was assigned to patients 2+ or 3+. Cytokeratin 5/6 expression was classified as positive when staining of >1% of the cells (cytoplasmic and/or membranous staining) [35]. Vimentin expression was classified as positive when >50% of the tumoral cells were stained. Focal positive staining was also recorded when positivity was restricted to certain areas of the tissue.

### Intrinsic subtype classification of TNBC patients

TNBC patients were stratified in Basal-Like (BL), Mesenchymal- Like (ML) and Non-BL/Non-ML (NonBLML). The classification of intrinsic subtypes was done according the expression of EGFR, cytokeratin 5/6 and vimentin by IHC. Patients with any degree of positive expression (even focal) for EGFR and/or cytokeratin 5/6 were classified as Basal-Like as proposed by Nielsen and coworkers [10]. Patients with negative EGFR and cytokeratin 5/6 expression and positive expression of vimentin were classified as Mesenchymal-Like. Vimentin is regarded as a major and conventional canonical marker of EMT [50] and Mesenchymal-like (Claudin-low) tumors have been described to show higher vimentin expression levels compared to Basal-like and other tumor subtypes [8, 44, 45]. Therefore, positive vimentin expression was used to classify Non-Basal TNBC patients as Mesenchymal-like. If negative expression was obtained for these three proteins, patients were classified as Non-BL/Non-ML.

### Statistical analysis

Continuous variables were expressed as mean ± standard and compared using unpaired t-test or Mann-Whitney U-test according to the data distribution with or without normality. Categorical variables were presented as the number and percentage in each category and were compared as needed using χ^2^ test or Fisher exact test. When appropriate, Bonferroni test was used as a post hoc comparison test. Mantel-Haenszel test for linear trend was used to examine the relationship for ordinal variables.

Patients without available information on post-diagnosis relapse or survival status within at least 5-years after diagnosis were not included in the survival analysis. Relapse information was obtained from clinical records. Survival status was obtained from the Hospital Dr. Josep Trueta Cancer Register. The cut-off point for defining survival status was set at 30^th^ September 2015. Overall survival (OS) was defined as the interval between the date of diagnosis and the date of patient death. Disease-free survival (DFS) was defined as the interval between the date of diagnosis and the date of patient’s first local or distant relapse. Survival curves were estimated using the Kaplan–Meier method and survival differences between groups were determined via the log-rank test. Cox proportional hazard model was used to examine the effect of several clinical and histological variables on survival outcomes. Results are shown with estimated hazards ratios (HRs) and their 95% confidence intervals (CI). A p-value of <0.05 was considered statistically significant. All statistical analyses were performed with SPSS version 23.0 data analysis [51] and R software [52].

## Abbreviations

95% CI: 95% confidence interval
BL: Basal-like
CK5/6: Cytokeratins 5/6
EGFR: Epidermal grow factor receptor
FASN: Fatty acid synthase
HR: Hazard ratio
IHC: Immunohistochemistry
IQR: Interquartile range
ML: Mesenchymal-like
OS: Overall-survival
DFS: Disease-free survival
TMA: Tissue microarrays
TNBC: Triple-negative breast cancer
